# Exploring the role of gelsolin and high-density lipoproteins in autoimmune diseases: spotlight on rheumatoid arthritis and systemic lupus erythematosus

**DOI:** 10.1093/oxfimm/iqag002

**Published:** 2026-01-25

**Authors:** Julián Pérez-Ocampo, Juan C Hernandez

**Affiliations:** Infettare, Facultad de Medicina, Universidad Cooperativa de Colombia, Medellín, 050016, Colombia; Infettare, Facultad de Medicina, Universidad Cooperativa de Colombia, Medellín, 050016, Colombia

**Keywords:** gelsolin, high-density lipoproteins, autoimmune diseases, rheumatoid arthritis, systemic lupus erythematosus, immunomodulation

## Abstract

Rheumatological systemic autoimmune diseases constitute a significant health problem globally due to their chronicity, potentially permanent incapacity and higher mortality rates. Rheumatoid arthritis and systemic lupus erythematosus are among the most common of these diseases. Although there is a substantial body of research on these autoimmune diseases, there is still a need to identify better biomarkers of disease activity and progression. In this context, gelsolin and high-density lipoproteins arise as novel biomarkers from the perspective of immunomodulation and their role in the immunopathology of autoimmune diseases. Gelsolin is an actin cytoskeleton remodeling protein involved in immune regulatory mechanisms related to inflammation. High-density lipoproteins are plasmatic cargo molecules involved in reverse lipid transport, whereas their study in autoimmunity has focused on their value as cardiovascular risk predictors. However, new functions of these proteins related to immune and inflammation regulation have also been described recently. Therefore, this review aims to provide insight into the role of these biomolecules and their implications in the immunopathology and immunomodulation of autoimmune diseases from the perspective of rheumatoid arthritis and systemic lupus erythematosus.

## Introduction

Autoimmune diseases constitute an important health problem in international settings. According to the World Health Organization (WHO), this disease affects between 3% and 5% of the global population, with growing numbers each year and a higher prevalence in females [1].

In this group of health disorders, rheumatological systemic autoimmune diseases are interesting due to their chronicity, potentially permanent incapacity and the higher mortality rates of the ailing compared to healthy individuals. In this respect, rheumatoid arthritis (RA), and systemic lupus erythematosus (SLE) are some of the most common diseases affecting the young female population [2].

Research on these diseases has focused mainly on three aspects: first, understanding their immunopathogenesis; second, the search for more effective therapies; and third, the exploration of new biomarkers of disease activity and prognosis [3, 4]. Although there is a robust body of investigations into the immunological characteristics of these disorders, their etiology has not been established. On the other hand, there have been significant advances in clinical therapies that have been effective in prolonging the life of rheumatological patients as well as their quality of life [5]. Last, even though many biomarkers have emerged over the years, this has not led to a precise estimation of disease activity or patient prognosis, so the need to search for new biomarkers is still an active field of research, particularly in SLE [6].

In this regard, biomolecules such as high-density lipoprotein (HDL) and gelsolin, whose effects on these diseases are not related to any immunological disorder, have attracted increasing interest because of their involvement in immunomodulation [7, 8]. This narrative review aims to highlight the recent panorama in the study of HDL and GSN and their impact in a rheumatic autoimmunological context from the perspective of rheumatoid arthritis and systemic lupus erythematosus.

## Gelsolin

Gelsolin is a protein with the capacity to interact with actin filaments. Its central involvement is in cytoskeleton restructuring through the nucleation, capping or severing of the actin cytoskeleton, which is implicated in other cell functions, such as chemotaxis, secretion and shape determination [9]. Genetically, its encoding gene is located on chromosome 9 in humans. It is composed of 730 amino acids with a molecular weight of 82–84 kDa, and these amino acids form six consecutive domains (G1–G6), each of which participates in biological activity. Gelsolin has two forms, a cytosolic form (cGSN) and a plasmatic form known as plasma gelsolin (pGSN), which only differ from cGSN by an additional 24 amino acid N-terminal extension [10]. Both can depolymerize actin filaments and are associated with actin scavenging and clearing of cellular debris [11].

### Biological roles of the Gelsolin

**Figure 1:**
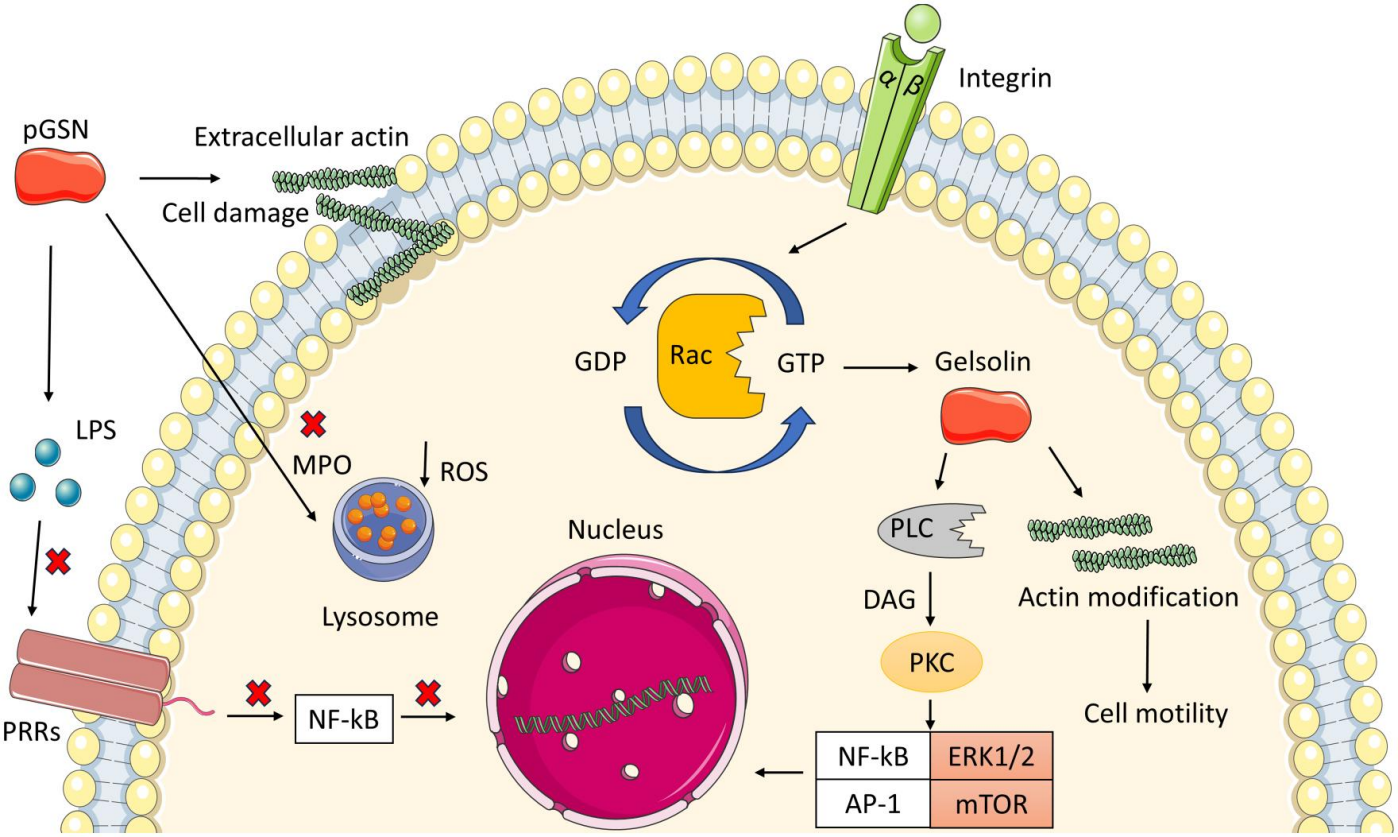
Plasma and intracellular gelsolin functions. Plasma gelsolin can bind microbial products such as LPS, reducing signaling via pattern recognition receptors (e.g. TLR4) and therefore reducing NF-κB inflammatory responses [16]. Moreover, pGSN can reduce cellular myeloperoxidase activity, diminishing reactive oxygen species production [11]. Furthermore, pGSN binds actin filaments derived from cell death, allowing them to be cleared by the mononuclear phagocytic system [17]. On the other hand, phagocytosis mediated by integrins can modulate cell motility via the GTPase rac [18] as well as several cellular pathways via protein kinase C, resulting in NF-κB and AP-1 translocation to the nucleus and activation of the ERK1/2 and mTOR pathways related to processes such as proliferation, survival, chemotaxis, actin remodeling, muscle contraction, metabolic regulation, and inflammatory epithelial responses, among others [19, 20]. pGSN: plasma gelsolin, LPS: lipopolysaccharide, PRRs: pattern recognition receptors, MPO: myeloperoxidase, ROS: reactive oxygen species, NF-kB: nuclear factor kappa B, GDP/GTP: guanosine di/triphosphate, PLC: phospholipase C, DAG: diacylglycerol, PCK: protein kinase C, AP-1: activator protein 1, ERK1/2: extracellular signal-regulated kinases, mTOR: mammalian target of rapamycin. The figure was partly generated using Servier Medical Art, which was provided by Servier and licensed under an unported creative commons attribution 3.0 license.

The isoforms of gelsolin are associated with many homeostasis-related functions. Guo and col. (2018) showed that GSN can regulate apoptosis in lymphoma NK cells from the YTS cell line and that endogenous GSN can reduce cell proliferation by promoting apoptosis via the PI3K/Akt signaling pathway [12]. In addition, Wang *et al.* (2023) reported that gelsolin inhibited SW620 colorectal cancer cells by promoting apoptosis via upregulation of the transcription of the death receptor-related pathway genes TNFR2 and CASP10 [13]. In contrast, Hong Zang *et al.* (2011) reported that intravenously injected GSN can reduce brain injury and inflammation after thermal damage in BALB/c mice by downregulating apoptosis by reducing caspase-3 expression and activity [14]. GSN is also involved in lipid metabolism by modulating phospholipase C (PLC) activity. PLC hydrolyzes phospholipids of the inner membranes of cells, mainly phosphatidylinositol-4,5-bisphosphate (PIP2), which then generates inositol-1,4,5-triphosphate (IP3) and diacylglycerol (DAG), which are second messengers that end in Ca2+ intracellular release and activation of protein kinase C (PKC) and are thus involved in numerous cellular processes, such as cell proliferation and survival, chemotaxis, muscle contraction, metabolic regulation, and inflammatory epithelial responses, among others [15] (Fig. 1). Another cellular process in which the GSN is involved is cellular motility signaling. It has been found that the cGSN is a necessary effector of cellular motility through Rac signaling. Rac is a GTPase from the rho family that transduces signals upon activation to achieve actin-based membrane ruffling [21] (Fig. 1). By using mouse dermal fibroblasts lacking gelsolin (Gsn^-^), the authors noticed a significant reduction in motility compared to that of wild-type cells. Moreover, they used the anti-Rac staining antibody and observed that the recruitment of Rac was appropriate in both cell lines. This suggests that the reduction in motility in Gsn^-^ cells is related to downstream signaling [18]. One of the most relevant processes directly involved in inflammatory responses modulated by GSN is phagocytosis. In brief, phagocytosis is a complex cellular process that involves the engulfment or uptake and degradation of microorganisms and damaged cells, as well as cellular debris, by professional phagocytes (macrophages/monocytes, dendritic cells, neutrophils, mast cells, etc.) [22]. Phagocytes play a fundamental role in the remodeling of connective tissues and maintaining adequate homeostasis in tissue repair and local inflammation [23]. GSN can mediate this process through different receptors, as it has been shown to be involved in phagocytosis mediated by Fcγ receptors and integrins [24] (Fig. 1).

## High-density lipoproteins

**Figure 2:**
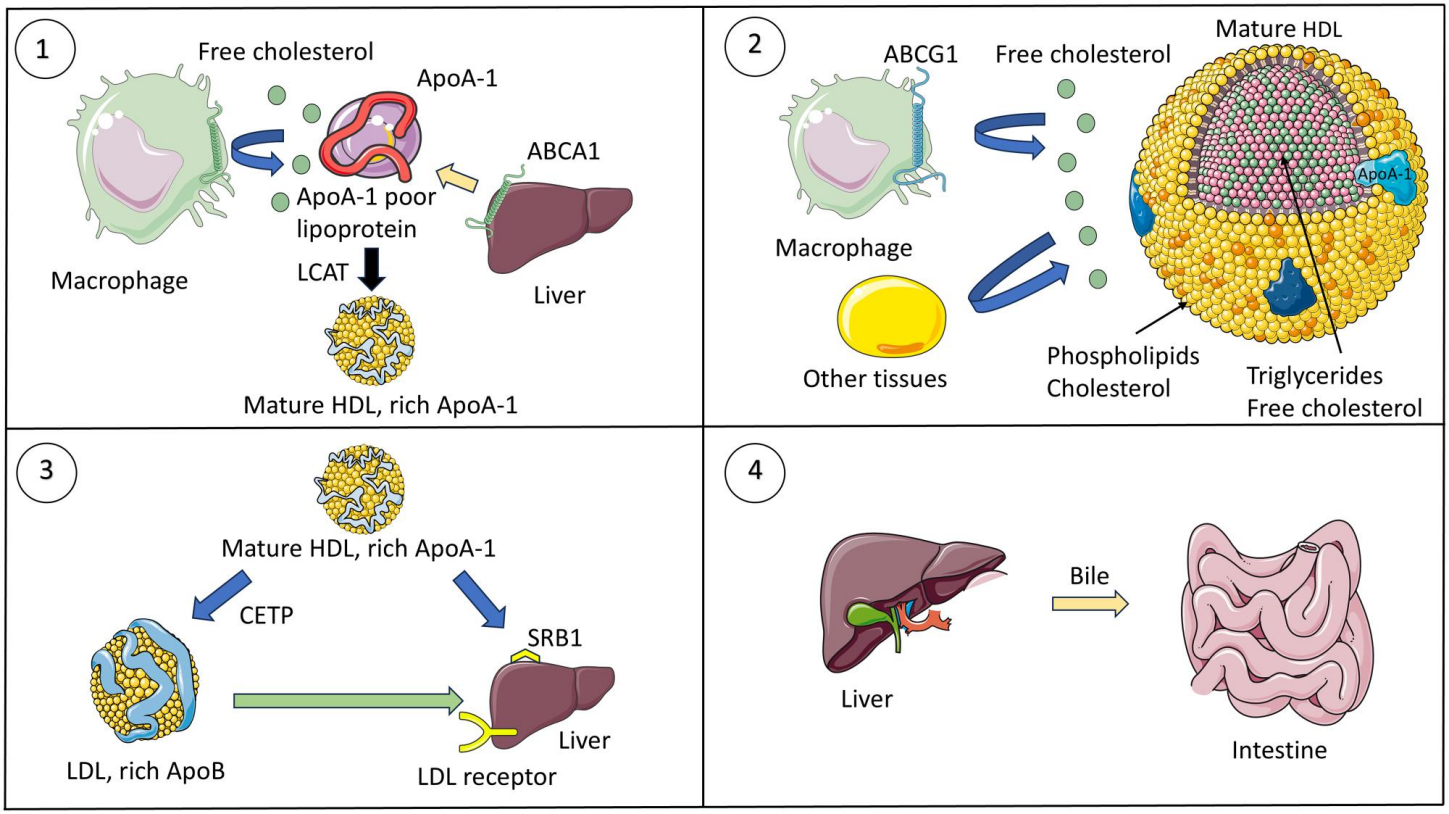
HDL maturation and reverse cholesterol transport. (1) HDL is synthesized in the liver and intestine and released into the circulation as ApoA-1 poor lipoproteins. These cells acquire phospholipids and cholesterol from hepatocytes through the ABCA1 transporter. Once in circulation, they undergo cholesterol efflux mainly from macrophages, which are then incorporated into nascent HDL. Once enough cholesterol is present, LCAT helps HDL to mature into ApoA1-rich lipoproteins. (2) Mature HDLs travel in circulation, charging themselves with excess cholesterol from cells and peripheral tissues. (3) Excess HDL cholesterol is transferred through CETP to ApoB-rich lipoproteins such as low-density lipoproteins or directly to hepatic cells via scavenger receptor B1. LDL also transfers cholesterol to the liver via LDL receptors. (4) Cholesterol can be secreted via the bile duct or used in hormone synthesis once in the liver. This figure was partly generated using Servier Medical Art, which was provided by Servier and licensed under an unported creative commons attribution 3.0 license.

High-density lipoproteins are plasma-circulating proteins responsible for carrying lipids in the circulation system, primarily cholesterol, from tissues to the liver for expulsion through the biliary pathway, a process known as cholesterol reverse transport (Fig. 2) [25]. Their structure is usually complex and comprises different particles varying in size and lipid composition. Its major constituents are cholesterol, esterified and free; phospholipids; apolipoproteins, namely, ApoA1, ApoA-II, ApoA-IV, ApoC, ApoE, ApoJ and ApoM [26]; and associated proteins, known as lecithin–cholesterol acyltransferase (LCAT), lipoprotein lipase (LPL) and cholesteryl ester transfer protein (CETP), which are implicated in HDL maturation and cholesterol reverse transport [27]. Moreover, HDL can have different particle subfractions that vary in size and composition. To date, two major HDL fractions have been described: HDL2, which is larger and lipid-rich, and HDL3, which is smaller and protein-rich. Furthermore, these fractions can be segregated into five subfractions [28]. Larger HDL have been shown to generate better cholesterol efflux and thus are associated with atheroprotective and anti-inflammatory effects [29]. Other enzymes, such as paraoxonase-1 (PON-1), are related to HDL and are responsible for some of its antioxidant and anti-inflammatory activities [30].

HDL has shown *in vitro* and *in vivo* capacity to bind to endotoxins and other bacterial products, causing less recognition by monocytes and macrophages and reducing cytokine secretion [31]. Norata *et al.* (2008) demonstrated that in the vascular endothelium, long-pentraxin 3 (PTX3) expression can be modulated by HDL, exerting anti-inflammatory and atheroprotective effects [32]. Moreover, ApoA1 could prevent lymphocyte activation and proliferation in peripheral lymph nodes in mice, as shown in the work of Wilhelm and colleagues (2009). In turn, a reduction in this proliferation is associated with fewer circulating antibodies and a reduction in inflammation, suggesting that this could be mediated by regulating the cellular cholesterol balance [33]. Furthermore, in inflammatory and atherosclerotic contexts, HDL-associated plasma platelet-activating factor-acetylhydrolase (PAFAH) has been shown to restore the migration of DCs from the skin to lymph nodes *in vitro* by inactivating PAF and oxLDL, thereby reducing plaque inflammation and size by clearing dead cells [31]. Lipid raft modification is a well-known mechanism associated with HDL immune modulation. Lipid rafts are cell membrane microdomains enriched with cholesterol and have a compact and differentiated structure comprising packed saturated glycosphingolipids and sphingomyelin [34]. These conserved microdomains limit protein membrane diffusion and concentrate these molecules within the lipid raft; this is associated with protein function in different cellular aspects, such as signaling or cytokine secretion [26]. An example of this immunomodulation is highlighted in the work of Koseki *et al.* (2007), who evaluated lipid raft involvement in TNF-alpha delivery to the plasma membrane and subsequent cellular secretion [35]. In this context, lipid rafts can cluster different Toll-like receptors (TLRs) and other cell membrane receptors, regulating the activation of specific signaling pathways, such as the TLR/MyD88 pathway, induced by LPS [36]. In this context, it has been suggested that HDL and APO-A1 can modulate the cholesterol present in lipid rafts through the ABC transporters ABCA1 and ABCG1, promoting cholesterol efflux (CE) from lipid rafts diminishing their signaling functions and therefore reducing the inflammatory response [31, 37].

Furthermore, pGSN can bind and thus modulate extracellular lipids and inflammation, although it does not directly build HDL, it helps maintain a healthy lipid transport environment. In this sense, pGSN can bind different modified apolipoproteins such as serum amyloid A and apolipoprotein H [11, 38], which are associated with higher levels of oxidized lipoproteins, particularly in the cases of SLE and RA. In contrast, HDL are altered and their anti-oxidizing activity is diminished [39, 40], they play an important role in the establishment of chronic inflammation and the promotion of autoimmunity. pGSN also binds sphingosine-1-phosphate (S1P), a bioactive lipid mediator involved in various cellular processes related to inflammatory and immune responses such as proliferation, migration, rearrangement of the cytoskeleton, and cell adhesion, among others [41]. Considering this, a reduction in pGSN leads to less effective clearance of inflammatory lipids, promoting lipid/LPS-induced inflammation that can modify HDL particles, impairing their reverse transport capabilities and reducing their cholesterol efflux capacity (CEC).

## Autoimmunity and rheumatological autoimmune diseases

**Figure 3:**
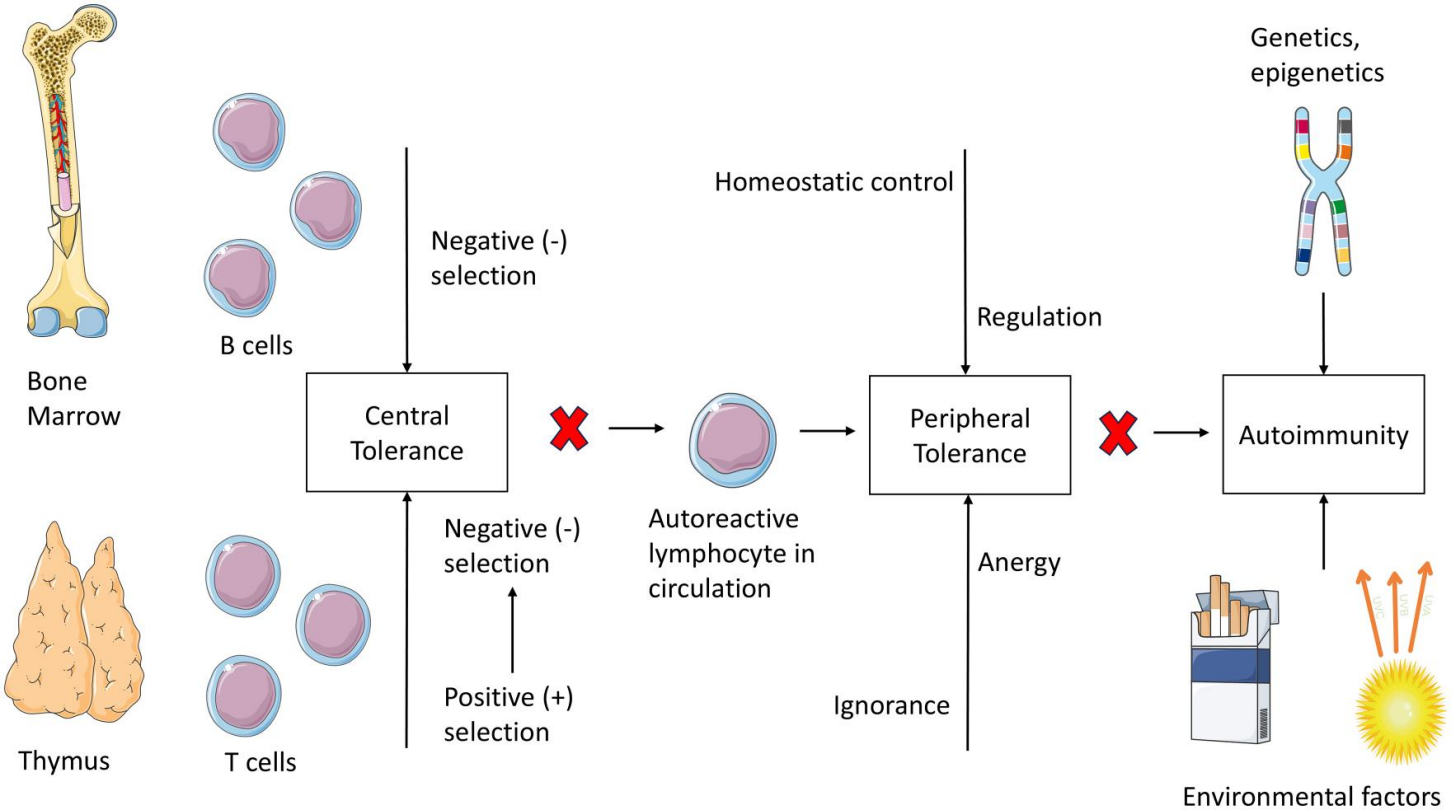
Central and peripheral tolerance mechanisms in autoimmunity. Lymphocyte maturation occurs in primary lymphoid organs (bone marrow and thymus). To induce central tolerance once the lymphocyte repertoire is selected, cells are subjected to positive selection where they must recognize self MHC-I or MHC-II through the T-cell receptor in the case of LTs. After this, negative selection is carried out, where either LTs or LBs that react strongly against self-molecules are deleted through apoptosis. On occasion, these selections fail, and autoreactive cells reach circulation. To address these dangerous cells, the immune system has several mechanisms known as peripheral tolerance. Four distinct effects have been described: 1: homeostatic control occurs when an activated T-cell expresses cytotoxic T-lymphocyte antigen 4 (CTL-4) or CD152 rather than CD28L; therefore, when a CD80/86 antigen-presenting cell interacts with CTL4, the T-cell is switched off. 2: Regulation mediated by specialized regulatory T cells can inhibit cytokine production or signaling pathways. 3: Anergy occurs when a lymphocyte receives an antigenic signal without the appropriate costimulatory second signal, which leads to a state of metabolic arrest, which can result in apoptosis. In the case of autoreactive cells, when these cells interact with self-peptides on parenchymal cells, the latter cannot provide an appropriate costimulatory signal. 4: Ignorance, this keeps autoreactive lymphocytes are ignored because of the concealment of autoantigens through cellular or epithelial barriers; thus, the autoimmune cell never reaches its antigen before undergoing apoptosis. Moreover, peripheral tolerance can fail, and autoimmunity can occur if autoantigen recognition occurs under conditions involving appropriate genetic, epigenetic, and environmental factors. The figure was partly generated using Servier Medical Art, which was provided by Servier and licensed under an unported creative commons attribution 3.0 license.

Autoimmunity is a phenomenon that occurs when an immune response occurs against self-molecules. It is mainly driven by a breakdown in immunologic tolerance to autoreactive immune cells [42]. The concept of immune tolerance comprises a set of mechanisms to help ensure the elimination of autoreactive immune cells [43]. Tolerance mechanisms can be divided into two categories: central and peripheral tolerance. Central tolerance mainly occurs after the generation of the lymphocyte repertoire in primary lymphoid organs (thymus or bone marrow). This is mediated by the negative selection of strong self-antigen-reacting lymphocytes, leading to their death via apoptosis; however, this mechanism can fail and thus, lead to autoreactive cells escape from this deletion. In this scenario, the peripheral tolerance mechanisms step into action, but again, these mechanisms can fail to delete these self-reactive cells either by interacting incorrectly with environmental factors or genes [44]. This process is illustrated and expanded upon in Fig. 3.

Once tolerance is disrupted, autoimmunity is established. Autoimmune diseases are pathological entities that are fundamentally supported by continuous autoimmunity phenomena. These diseases include a wide plethora of disorders and symptoms that can be organ-specific or systemic. An interesting characteristic of these diseases is the lack of a clear etiology, whereas a combination of genetic, epigenetic and environmental factors drives the inception of the disease [45]. Rheumatoid arthritis, systemic lupus erythematosus, thyroiditis, scleroderma, multiple sclerosis and diabetes mellitus type I are classically the prototypes of autoimmune diseases [46]. Nonetheless, other pathological disorders, such as atherosclerosis, schizophrenia, and inflammatory bowel disease, among others, can exhibit autoimmunity even when autoimmune diseases are not present [47].

As autoimmune diseases, SLE and RA share some immunological and clinical characteristics. First, they present with immunological tolerance breakdown, leading to immune responses against self-antigens with the generation of autoantibodies against different cellular components, which is their most remarkable characteristic [3, 48].

### Rheumatoid arthritis

Rheumatoid arthritis (RA) is an autoimmune disease characterized by systemic activity, chronic inflammation, and joint pain. [49]. Its etiology primarily involves genetic factors, particularly polymorphisms in HLA type 2 genes, which significantly increase the risk of developing the condition [50]. However, the manifestation of RA necessitates interactions between an individual’s susceptible genotype and environmental and risk factors (e.g. smoking tobacco) [51]. Immunologically, RA is marked by the production of autoantibodies, notably rheumatoid factor—a type of autoantibody targeting the Fc portion of IgG immunoglobulins—and autoantibodies against citrullinated self-proteins [52]. The continuous production of these autoantibodies, coupled with the accumulation of cytokines and other inflammatory mediators, contributes to the chronic inflammation commonly observed in joints [53].

### Systemic lupus erythematosus

Systemic lupus erythematosus (SLE) is an autoimmune systemic disorder that imposes a chronic burden on affected individuals. It presents a broad spectrum of clinical symptoms, from skin rashes to compromised organ functionality. While SLE can affect anyone, premenopausal women constitute the most commonly affected population [54]. The immunopathogenesis of SLE is well understood, although its definitive etiology remains elusive. Susceptible genotypes, such as certain HLA class II haplotypes (HLA-DR2 and HLA-DR3), and homozygotic deficiencies in complement elements, notably C2 and C4, increase the relative risk of developing SLE [55]. However, this predisposition alone is insufficient for disease manifestation. Increased exposure to autoantigens, typically nucleic acids, occurs initially, possibly triggered by acute inflammation from trauma, infections, or prolonged ultraviolet radiation. This leads to cellular death and subsequent release of stimulating autoantigens [56]. In the presence of a breakdown in immunotolerance mechanisms, autoreactive immune cells recognize these autoantigens, initiating an autoimmune response. Additionally, innate immune responses, driven by type I interferons, particularly IFN-α produced by plasmacytoid dendritic cells, potentiate various cellular effects, including autoreactive T-cell proliferation and activation, antigen presentation, and B-cell differentiation into autoantibody-secreting plasma cells, ultimately establishing autoimmunity [57]. Pathologic autoantibodies circulating in the blood can recognize self-molecules in various tissues, forming immune complexes that deposit in the tissue endothelium, amplifying immune responses through inflammation by stimulating proinflammatory cytokine production, reactive oxygen species production, and complement activation, directly causing tissue damage [58].

## Gelsolin and high-density lipoproteins in autoimmune diseases

**Figure 4:**
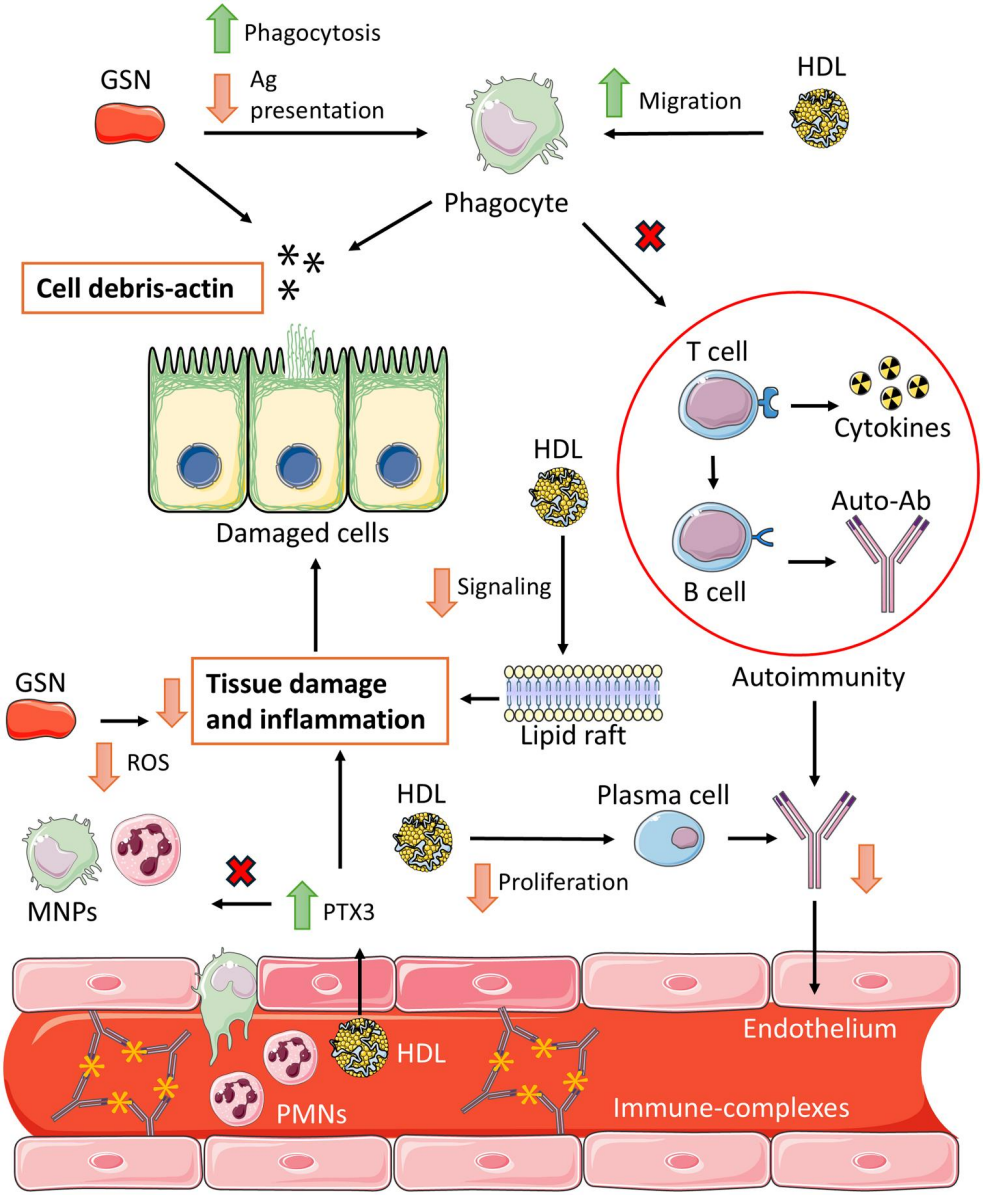
Proposed mechanisms of GSN and HDL immunoregulation in autoimmunity. GSN and HDL can modulate autoimmunity in two principal ways: first, they can enhance cellular debris clearance and, therefore, remove potential sources of autoantigens, diminishing antigen presentation to T cells and preventing autoantibody generation. pGSN can bind extracellular actin residues intertwined with cellular molecules and promote their phagocytosis by mononuclear phagocytes, while cGSN regulates phagocyte motility and phagosome formation [7, 17]. HDL promotes the migration of monocytes, enhancing cellular debris clearance [60]. Second, they can act as regulators of inflammation in consequent damage by immunocomplex deposition in tissues, reducing cellular death by inflammatory mechanisms limiting autoantigen exposure. pGSN can regulate reactive oxygen species generation by polymorphonuclear cells and mononuclear phagocytes [11], which dampens oxidative stress and potential tissue damage through excessive ROS production. On the other hand, HDL modulates inflammatory mediators by reducing receptor clustering and inflammatory pathway signaling via lipid raft modification [35, 37]. HDL upregulates the expression of PTX3, which modulates inflammatory responses and reduces immune cell infiltration in the endothelium [32]. HDL regulates lymphocyte activation and proliferation, decreasing the production of circulating autoantibodies [33]. GSN: gelsolin, pGSN: plasma gelsolin, cGSN: cytosolic gelsolin, HDL: high-density lipoprotein, Ag: antigen, Ab: antibody, PMNs: polymorphonuclears, MNPs: mononuclear phagocytes, PTX3: pentraxin-related protein 3. This figure was partly generated using Servier Medical Art, which was provided by Servier and licensed under an unported creative commons attribution 3.0 license.

The plasma levels of gelsolin, which are usually measured through immunoassays or western blotting, have been investigated in inflammatory contexts, primarily in infectious diseases [9, 16, 59]. As stated before, GSN is important for modulating and regulating inflammation. This interesting biomolecule, however, has only recently been gaining attention in other inflammation-bearing disorders, such as autoimmune diseases. A similar situation occurs with HDL. This section aims to briefly describe what researchers have found regarding GSN and HDL and inquire how studying these molecules would help change our perspective of autoimmune diseases. Moreover, it is important to note that immune-mediated mechanisms differ, led by activation and molecular pathways in acute inflammatory processes such as trauma, sepsis, infection, and acute respiratory distress syndrome (ARDS), among others. Regarding this, it has been described that in acute inflammatory processes, the signaling pathways start by the surge of pathogen-associated molecular patterns (PAMPs) or damage-associated molecular patterns (DAMPs), these molecules are then recognized by pattern recognition receptors present in immune cells, mainly Toll-like receptors (TLRs) whom down-signal through MyD88/TRIF pathways, which lead to the activation of nuclear factor kappa B (NF-κB), mitogen-activated protein kinases (MAPK) and the Janus kinase/signal transducer and activator of transcription (JAK/STAT). This leads to the promotion of inflammatory responses through cytokines, chemokines, and cell adhesion molecules [11, 14, 16]. Meanwhile, in chronic inflammatory contexts, signaling starts by the presence of autoantigens in immune complexes and involves TLRs, JAK–STAT, MAPK and cGAS–STING signaling pathways (Fig. 4).

### Gelsolin levels in autoimmune diseases

Plasma GSN levels are usually lower in autoimmunity-bearing patients, although further evidence of the relationships between these levels and patients’ clinical and immunological status is needed. Nevertheless, several researchers have explored this association in animal models and humans. The features of most of these original studies published to date are summarized in Table 1.

**Table 1:** Summary of original investigations about GSN levels in autoimmune diseases.

Year	First author	Study type: model	Disease	GSN levels	Reference
1993	Weeds, A. G	In vivo: Human	Familial amyloidosis	Lower in blood	[61]
2005	Aidinis, V.	In vivo: gsn-/- Tg197 mice	Rheumatoid arthritis	N/A	[62]
2008	Osborn, T. M.	In vivo: Human	Rheumatoid arthritis	Lower in blood	[62]
2008	Kulakowska, A.	In vivo: Human	Multiple sclerosis	Lower in CSF	[63]
2010	Kulakowska, A.	In vivo: Human	Multiple sclerosis	Lower in blood and CSF	[64]
2013	Hu, YL.	In vivo: Human	Rheumatoid arthritis	Lower in blood	[65]
			Systemic lupus erythematosus		
2014	Genre, F.	In vivo: Human	Ankylosing spondylitis	Lower in blood	[67]
2015	Argun, M.	In vivo: Human	Acute rheumatic fever	Lower in blood	[68]
2015	Gungor, H. E.	In vivo: Human	Atopic dermatitis	Lower in blood	[69]
2016	Hsieh, LC. K.	In vivo: SJL mice	Multiple sclerosis	Lower in blood	[22]
				Higher in brain	
2020	Esawy, M. M.	In vivo: Human	Psoriasis	Lower in blood	[70]
2020	Parra, S.	In vivo: Human	Systemic lupus erythematosus	Lower in blood	[71]
2021	Huang, H.	In vivo: Human	Sjogren’s syndrome	Lower in blood	[72]
2022	Mosaad, G. M.	In vivo: Human	Systemic lupus erythematosus	Lower in blood	[73]
2022	Hamdino, M.	In vivo: Human	Atopic dermatitis	Lower in blood	[74]

CSF: cerebrospinal fluid.

Weeds *et al.* (1993) described a GSN genetic variant related to familial amyloidosis, which presented with lower levels of this protein and reduced actin severing and nucleating capabilities, indicating an association with disease immunopathology [61]. Aidinis and collaborators (2005) conducted an *in vivo* study analyzing how the suppression of GSN gene expression could affect an RA mouse model. They mated gsn^-/-^ mice with Tg197 arthritic mice and found that the resulting offspring had exacerbated disease with synovial membrane hyperplasia and inhibition of the actin-severing activity of gelsolin [66]. Osborn and colleagues (2008) reported that in a cohort of rheumatoid arthritis patients, pGSN levels were significantly lower than those in sex- and age-matched healthy controls [62]. These authors associated these lower levels in RA patients with a depletion of pGSN due to local consumption in joint inflammation. Kulakowska *et al.* (2008) reported lower GSN concentrations in the cerebrospinal fluid (CSF) of patients with multiple sclerosis (MS) than in controls without CSF alterations; these levels were also associated with a lower actin-severing capability, suggesting an explanation for some of this disease’s pathological characteristics [63]. They later found in another study (2010) that these GSN levels were also decreased in the blood of MS patients [64], indicating direct participation of GSN between chronic inflammation and neurodegenerative damage in this disease. Hu *et al.* (2013) evaluated pGSN in patients with RA and SLE; in both groups, they observed significantly lower levels of this protein in patients’ plasma than in healthy controls. Interestingly, they also found that RA patients had lower levels of pGSN than did SLE patients and that in the case of SLE, pGSN negatively correlated with disease activity. However, this was not the case for the RA cohort [65]. Genre and col. (2014) also reported decreased pGSN levels in patients with ankylosing spondylitis (AS) undergoing biological immunotherapy with anti-TNFα antibodies compared to age- and sex-matched controls. Notably, the group of AS patients they assessed had adequate control of the disease [67]. Argun and others (2015) described a cohort of patients with acute rheumatic fever with lower pGSN levels than healthy controls, which were also correlated with a poorer prognosis of carditis [68]. Gungor *et al.* (2015) reported lower pGSN levels in a cohort of pediatric patients with atopic dermatitis than in healthy controls who were age- and sex-matched. They also found that the pGSN correlated with disease severity [69]. Hsieh and colleagues (2016) performed an *in vivo* study with a mouse model of experimental autoimmune encephalomyelitis (EAE) and found that these mice had decreased GSN levels in the blood. Moreover, they intravenously administered recombinant human plasma gelsolin to the mice. They observed a reduction in extracellular actin as well as a reduction in myeloperoxidase activity in the brain, reflecting a decrease in disease activity and severity [17]. Esawy and collaborators (2020) reported lower pGSN levels in a group of patients with psoriasis than in healthy controls. This study also evaluated a cohort of psoriasis patients with psoriatic arthritis. They observed that the levels of pGSN were even lower than those of psoriasis patients without articular compromise, suggesting a relationship with joint inflammation [70]. Huang and col. (2021) studied the pGSN in patients with primary Sjogren’s syndrome (pSS) and reported lower levels of this biomolecule in the serum of pSS patients than in healthy controls. Furthermore, they also observed a reduction in the expression of GSN in whole blood cells; however, they found no correlation between disease activity and GNS levels in those patients [71]. Mosaad *et al.* (2022) assessed a cohort of fifty SLE patients and found decreased levels of pGSN in those patients compared to healthy controls. They also found a strong negative correlation between GSN levels, disease activity and organ damage. Notably, they suggested that pGSN levels lower than 78.95 μg/ml could be used as a predictor of renal damage in patients with SLE [72]. Hamdino and others (2022) described significantly lower levels of pGSN in a cross-sectional study of atopic dermatitis in pediatric and adult patients than in healthy controls. Interestingly, they found no differences between children and adults but observed correlations with disease severity as well as other clinical markers, suggesting that pGSN is involved in disease progression independent of age [73]. Although, the studies we presented in this review did not considered longitudinal evaluations of pGSN levels in these autoimmune diseases, it is important to note that dynamic changes in these may occur differently in each disease and should be assessed individually; as is it known that under other inflammatory/disease conditions such as trauma, sepsis, fibrosis, liver injury, etc. these pGSN vary according to the clinical course, being that a reduction associates with poorer prognosis and evolution and a restoration is related to a reduction in negative clinical outcomes such as death [74–76].

### High-density lipoproteins in autoimmune diseases

High-density lipoproteins have a long history in autoimmune diseases; these biomolecules have been of particular interest in assessing cardiovascular risk in rheumatological patients, as almost every autoimmune disease is related to increased cardiovascular diseases mediated by inflammation, such as atherosclerosis, this has been explored by Camont *et al.* [77] and Kontush *et al*. [78]. Altered HDL is associated with quantitative and qualitative changes that impair cholesterol efflux [79]. Moreover, it is widely accepted that the structure and associated enzymes of high-density lipoproteins are modified because of chronic inflammation in these disorders. It has mainly been described that HDL loses Apo-A1, resulting in the production of apolipoprotein serum amyloid A (SAA) and impairing the anti-inflammatory and antioxidant activity of HDL; this HDL is currently known as proinflammatory HDL (piHDL) [80]. This piHDL ultimately reduces cholesterol efflux across tissues and cells, leading to increased inflammation and dysregulation of immune responses [81]. Many *in vitro* and *in vivo* studies have shown these inflammatory HDL disruptions in autoimmunity models and inflammatory contexts [82–89]. However, there is still limited evidence in human epidemiological research on autoimmune diseases.

An interesting study performed in Denmark by Madsen *et al.* (2019) showed how low HDL levels can promote the development of autoimmune diseases. They included two groups of healthy participants from two previous studies in Denmark. The first group was derived from the Copenhagen general population study that started in 2003. It comprised 107 954 individuals, and the second group came from the Copenhagen Heart Study, which started in 1997 and included 9387 individuals with a baseline measurement of HDL. They followed up in 2017 and found that 4078 participants from the first study and 1101 from the second study developed an autoimmune disease. Interestingly, in the groups that developed the disease, the adjusted hazard ratio for autoimmune diseases was significantly greater in those with the lowest HDL levels (<39 mg/dL). This indicates that the dysregulation of immunity mediated by HDL is associated with autoimmunity, although no causality could be established [90].

On the other hand, there is a robust body of HDL levels in autoimmunity-bearing patients. In contrast, most of these studies show reduced HDL levels in patients compared to controls, which demonstrates that there are at least quantitative abnormalities in the HDL levels of autoimmune patients [91–95]. Some studies that have evaluated HDL in autoimmune patients, focusing on its possible role as an immunomodulator, are summarized in Table 2 and discussed next.

**Table 2:** Summary of original investigations about HDL levels in patients with autoimmune diseases.

Year	First author	Study type: model	Disease	HDL levels	Reference
2005	Borba, E. F.	In vivo: human	Systemic sclerosis	Lower	[97]
2006	McMahon, M.	In vivo: human	Systemic lupus erythematosus	piHDL	[98]
			Rheumatoid arthritis		
2009	Charles-Schoeman, C	In vivo: human	Rheumatoid arthritis	piHDL	[99]
2010	McMahon, M.	In vivo: human	Systemic lupus erythematosus	piHDL	[100]
2011	Popa, C.	In vivo: human	Rheumatoid arthritis	Lower	[101]
2012	Charles-Schoeman, C	In vivo: human	Rheumatoid arthritis	Lower	[102]
2012	Holzer, M.	In vivo: human	Psoriasis	Lower	[103]
2012	Mehta, N. N.	In vivo: human	Psoriasis	Lower	[104]
2013	Ronda, N.	In vivo: human	Systemic lupus erythematosus	Lower	[105]
			Rheumatoid arthritis		
2016	Gaál, K.	In vivo: human	Systemic lupus erythematosus	Lower	[106]
2020	Parra, S.	In vivo: human	Systemic lupus erythematosus	Lower	[71]
2021	Ferraz-Amaro, I.	In vivo: human	Systemic sclerosis	Lower	[107]

piHDL: proinflammatory high-density lipoprotein.

A series of observational studies by McMahon and colleagues (2006, 2009, 2010) carried out on various cohorts of SLE and RA patients revealed the importance of piHDL in rheumatic diseases. In their first work, they assessed 150 SLE patients alongside 48 RA patients; in both groups, they found decreased HDL levels but increased piHDL levels compared to healthy controls. Noticeably, they found that the frequency of piHDL was greater in the SLE group. Moreover, they analyzed the levels of oxidized LDL, which correlated with piHDL levels [96]. The second study included 276 women with SLE. This study revealed that piHDL could be used as a precise predictor of cardiovascular risk [97]. A third study of 132 RA patients revealed that piHDL is correlated with disease activity, systemic inflammation and decreased HDL-associated enzyme activity [98]. These studies strongly indicate that HDL are important in SLE and RA immunopathology beyond quantifiable alterations and highlight the importance of assessing all dimensions of HDL functionality. Popa *et al.* (2011) examined lipids and PON-1 activity before and after anti-TNF therapy in a cohort of 45 plasma RA patients. They found that HDL levels were relatively low, and PON-1 activity increased, accompanied by a reduced inflammatory status. This could indicate that the immunomodulatory effects of HDL can indirectly be related to tumor necrosis factor activity, revealing an interesting focus on the immunomodulatory effect of the HDL innate immune response [99]. A study by Charles-Schoeman and col. (2012) In RA patients, cholesterol efflux capacity (CEC) was assessed due to HDL; however, they did not separate ApoB-rich lipoproteins. Nonetheless, decreased CEC correlated with decreased HDL levels, decreased antioxidant capacity, increased disease activity, reduced PON-1 activity and increased myeloperoxidase (MPO) activity [100]. Holzer and colleagues (2012) performed a study in psoriasis patients and reported that HDL levels, HDL antioxidant capacity and PON-1 activity were lower in psoriasis patients than in healthy controls, which also led to an inflammatory clinical profile in psoriasis patients [101]. Mehta *et al.* (2012) also evaluated psoriasis patients and reported similar results. Moreover, they examined lipoprotein composition and observed a profile similar to that of people with diabetes related to chronic inflammation [102]. Another study by Ronda *et al.* (2013) on RA and SLE patients evaluated cholesterol efflux capacity in ApoB-poor serum. They observed lower levels of HDL in both RA and SLE patients than in healthy controls, which was correlated with lower cholesterol efflux; they also found that this lower CEC was correlated with clinical inflammatory markers and disease activity [103]. Gaál *et al.* (2016) conducted a study in SLE patients and reported markedly lower levels of HDL and Apo-A1 than in healthy controls. Furthermore, they also assessed PON-1 activity and HDL antioxidant capacity, which were also lower in the SLE group. They also found a positive correlation between inflammatory markers and disease activity, and interestingly, HDL and antioxidant capacity were negatively correlated with these markers [104]. This indicates that inflammation in patients with SLE induces modifications in HDL composition and quantity, which could impair the regulation of inflammation by HDL. Ferraz-Amaro and col. (2021) performed a study in systemic sclerosis patients in which they not only found lower levels of HDL than in healthy controls but also observed lower ApoA-1 and cholesterol efflux; nonetheless, they found no correlation between these measurements and skin thickness, indicating independence from disease activity [105]. Borba *et al.* (2005) also evaluated an SSc cohort but focused on lcSSc; they also observed lower HDL in patients. Interestingly, they found that these reduced levels were correlated with the presence of anti-centromere autoantibodies; however, they did not encounter any other correlation with other markers [106].

An interesting study by Parra *et al.* (2020) involving *in silico* analyses of the proteome of purified HDL from 9 SLE patients before and after disease onset revealed that gelsolin was one of the proteins with the greatest fold change in expression. They proceeded to evaluate HDL and pGSN levels in a cohort of 104 SLE patients and found that in all of them, both biomolecules levels were decreased compared to those in healthy controls; moreover, this reduction was greater in patients in a clinical flare. Furthermore, they found that HDL cholesterol and pGSN were positively correlated, indicating that lower HDL levels are related to lower pGSN in patients with SLE [107].

### Limitations and prospectives

Although there are a handful of studies on RA and SLE that have consistently shown a significant reduction in pGSN levels in these two diseases and, in some cases, a correlation with clinical and immunological characteristics, particularly disease activity and severity, which are related to immune dysregulation, there is still limited knowledge of the immunomodulation mechanisms of GSN in the autoimmune context (other than its well-described anti-inflammatory activity), where GSN, either cellular or plasmatic, can directly immunomodulate adaptative immune responses or other innate immune mechanisms. There is still room for research in both animal models and humans that can expand our comprehension of the role of GSN in these two rheumatic diseases; transcriptomics could provide a powerful foundation for epidemiological and animal model studies in search of possible cellular and molecular mechanisms. Also, it is important to promote longitudinal studies on this molecule in RA and SLE, as this could further enhance our insight on the dynamics and effects of this protein alteration, which could prove useful in determining whether restoration to normal levels can be beneficial from a therapeutic perspective. This ultimately will lead to an exciting focus on gelsolin, which may open new possibilities for an attractive therapeutic target.

On the other hand, these studies indicate that HDL plays an important role in autoimmune diseases and has been studied in RA and SLE patients. In contrast, in some other autoimmune diseases, literature is limited. Although there are far more studies other than those presented in this review, most of them are focused on assessing HDL as a cardiovascular biomarker. Only a handful of these investigations have explored the association between HDL, an immunomodulator, and the three rheumatic diseases discussed here. This reveals the need to expand the study of HDL in autoimmune diseases, including new approaches incorporating clinical inflammation markers that could be related to the immunomodulatory capabilities of HDL. To date, it is widely accepted that HDL participates mainly in innate immune system regulation through the modulation of inflammatory responses. However, new *in vitro* and *in silico* evidence suggests that the adaptative immune response is to some extent directly or indirectly modulated by HDL and its associated proteins through poorly understood mechanisms. New approaches to *in-silico* studies and epidemiological research on human ailing populations will enhance our understanding of HDL beyond atherosclerosis in RA and SLE patients.

Regarding the use of GSN as a therapeutic agent, there have been some executed clinical trials focused primarily on acute damage and inflammation contexts. In the completed clinical trials, the NCT04358406 trial by BioAegis Therapeutics Inc, carried out between 2020 and 2022 in Romania and Spain, evaluated the efficacy, security and tolerability of intravenous (IV) doses of recombinant pGSN (rhu-pGSN) in SARS-CoV-2 patients with pneumonia, the results showed no significant adverse effects of rhu-pGSN compared to placebo, indicating its safe therapeutical use, however, no benefit regarding disease improvement was demonstrated for rhu-pGSN relative to placebo [108]. The NCT05789745 trial, also conducted by BioAegis Therapeutics Inc., took place in the United States of America (USA) in 2023. Its objectives were similar to those of the previous trial described, but it included healthy volunteers. Again, adverse events were not significantly higher in patients with IV rhu-pGSN compared to the placebo. The NCT03466073 trial, conducted by BioAegis Therapeutics Inc. in Australia in 2019, evaluated the effect of rhu-pGSN in individuals hospitalized for Acute Community-acquired Pneumonia (CAP), revealing the safety of using rhu-pGSN in these patients and highlighting the importance of trials with wider populations to assess clinical efficacy [109]. The NCT00671307 trial, conducted by Critical Biologics Corporation, also evaluated rhu-pGSN safety in hospitalized patients with documented low pGSN levels. Although the trial was conducted in 2010 in China, no results were achieved or posted.

On the other hand, three clinical trials are ongoing. The NCT05947955 trial by BioAegis Therapeutics Inc. aims to assess efficacy and safety in individuals with moderate to severe acute respiratory distress syndrome (ARDS) and is currently recruiting volunteers across the USA and Europe. The NCT06216366 trial by BioAegis Therapeutics Inc. aims to assess the capabilities of rhu-pGSN to regulate the inflammatory responses generated by decompression following diving in deep water in SCUBA divers, the researchers propose to evaluate different inflammatory markers such as IL-1β and others in groups receiving the recombinant pGSN and control groups, this trial is due to start its recruitment phase. Although the last active trial, NCT06712238 by Aswan University (Egypt), does not directly evaluate pGSN, its intervention objective is closely related to GSN. In this way, the trial aims to assess the effect of methotrexate, an immunosuppressive drug commonly used to treat and control autoimmune diseases, including RA and SLE, on the levels of pGSN in patients with psoriasis, a chronic immune-mediated skin affliction considered an autoimmune disease by most scholars and clinicians.

In light of these clinical trials, it is clear that gelsolin in its plasmatic form is safe as a therapeutic agent, unfortunately, it efficiency to clinical contribution both in acute inflammatory and chronic autoimmune context remains to be further demonstrated, as there is a lack of clinical trials focused on the effects of pGSN regarding inflammatory regulation in these conditions, especially in autoimmune diseases.

## Conclusion

Although gelsolin and HDL are well known for a long time to be involved in healthy homeostasis, their participation in immune pathologies is a relatively new approach. A growing body of scientific evidence connects these two important biomolecules with the regulation of the immune system; in this respect, both are involved in the regulation of the innate immune system and act primarily as anti-inflammatory molecules. This review summarizes these novel functionalities of GSN and HDL and contextualizes them from the perspective of autoimmune diseases, specifically rheumatoid arthritis and systemic lupus erythematosus, through *in vivo* studies of human patients. Therefore, the investigations presented here show that GSN and HDL are altered quantitatively and qualitatively in the two autoimmune diseases addressed in this paper. In the case of GSN alterations and their participation in promoting disease activity, severity and progression are evident, at least in RA and SLE patients. However, in others autoimmune diseases such as systemic sclerosis, there is still a knowledge gap. The role of HDL in disease activity and severity is well documented for individuals with RA. For SLE patients, there is still a need for a more immunologic orientation that strongly supports the implications of these molecules in the immunopathology of these two rheumatic diseases. Last, only one study provided the first step in exploring both GSN and HDL in an autoimmune context, specifically, SLE; however, this study alone is not enough to see a possible interplay between GNS and HDL. This ultimately reveals that the nature of the interaction between these two novel immunomodulators in autoimmune diseases is yet to be understood.

## Data Availability

All data generated or analyzed during this study are included in this published article.
